# Antibody-Drug Conjugates to Promote Immune Surveillance: Lessons Learned from Breast Cancer

**DOI:** 10.3390/biomedicines12071491

**Published:** 2024-07-05

**Authors:** Sabrina Nucera, Chiara Conti, Federica Martorana, Brooke Wilson, Sofia Genta

**Affiliations:** 1 Department of Human Pathology “G. Barresi”, University of Messina, 98131 Messina, Italy; sabrinanuc@libero.it (S.N.); conchiara9@gmail.com (C.C.); 2 Department of Clinical and Experimental Medicine, University of Catania, 95123 Catania, Italy; federica.martorana@unict.it; 3 University Oncology Unit, Humanitas Istituto Clinico Catanese, 95045 Misterbianco, Italy; 4 Department of Oncology, Queen’s University, Kingston, ON K7L 3N6, Canada; brooke.wilson@kingstonhsc.ca; 5 Division of Cancer Care and Epidemiology, Queen’s Cancer Research Institute, Kingston, ON K7L 3N6, Canada

**Keywords:** antibody-drug conjugates, immune-checkpoint inhibitors, immune surveillance, breast cancer, clinical trials

## Abstract

Antibody-drug conjugates (ADCs) represent an effective class of agents for the treatment of several tumor types, including breast cancer (BC), featuring approved molecules such as trastuzumab-emtansine, trastuzumab-deruxtecan, and sacituzumab-govitecan. Immune-checkpoint inhibitors (ICIs) also showed activity in selected BC subtypes, and two agents, pembrolizumab and atezolizumab, are currently approved for the treatment of triple-negative BC patients. The potential synergy between ADCs and immunotherapy in BC remains an area of active investigation. Preclinical studies suggest that ADCs promote immune surveillance, modulating tumor microenvironment, inducing immunogenic cell death, and enhancing antitumor immunity. Translational evidence has shown potential predictive biomarkers for ADCs alone or in combination with immunotherapy, including expression of target antigen, oncogenic pathways, tumor-infiltrating lymphocytes, and neutrophil-to-lymphocyte ratio. Given this background, several clinical trials evaluated ADC-ICI combinations in BC patients, demonstrating promising outcomes with an overall manageable toxicity profile, and many studies are currently ongoing to confirm the efficacy and feasibility of this therapeutic approach. In the present review, we summarized the available evidence about the integration of ADCs and immunotherapy for the management of BC, emphasizing the need for further translational and clinical investigations to optimize this treatment strategy and elucidate predictive biomarkers, eventually improving patient outcomes.

## 1. Introduction

**Figure 1:**
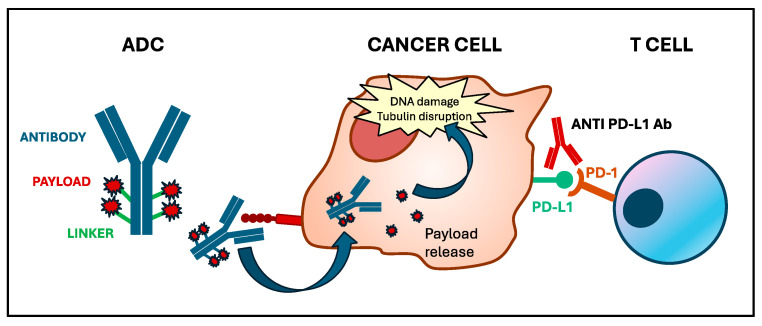
Mechanism of action of antibody-drug conjugates and immune-checkpoint inhibitors. ADCs consist of an antibody linked to a cytotoxic payload via a chemical linker. The antibody binds to a specific antigen on cancer cells, leading to internalization. Inside the cell, the linker is degraded, realizing the payload. This release causes DNA damage, leading to tumor cell death. ICIs, such as anti-PD-L1 antibodies, inhibit the bond between PD-L1 on tumor cells and PD-1 on T cells, allowing patients’ immune system to recognize and react against cancer cells.

Antibody-drug conjugates (ADCs) are powerful and versatile anticancer agents, with proven efficacy across multiple cancer types [1]. ADCs are comprised of three main components: a monoclonal antibody, a linker, and a drug payload [1]. Specific features of each of these components influence the efficacy and toxicity profile of ADCs [2]. The antibody binds to target antigens on the cancer cell surface, leading to internalization of the ADC, cleavage of the linker, and ultimately release of the payload, triggering tumor cell death. Additionally, the cytotoxic effect exerted by the payload is not the only mechanism contributing to the anticancer activity of ADCs. Mounting evidence indicates that these agents can modify the tumor microenvironment (TME), influence the interaction between cancer cells and the immune system, and thus have a complementary mode of action with immune-checkpoint inhibitors (ICIs) (Figure 1) [2,3]. These observations generate the following several questions: How crucial is the immunoregulatory activity of the ADCs in achieving treatment response? Can biomarkers classically used for immunotherapy have a role in predicting the benefit from ADCs? Do ADCs and ICIs have a synergistic effect?

With the first ADC approved for use in breast cancer (BC) more than 10 years ago, BC is the ideal setting to try to answer some of these questions. The large amount of data generated from clinical studies and real-world analyses in BC offers a unique opportunity to shed some light on the immunostimulatory potential of ADCs.

## 2. ADCs and Immunotherapy: Current Approvals for BC Patients

**Figure 2:**
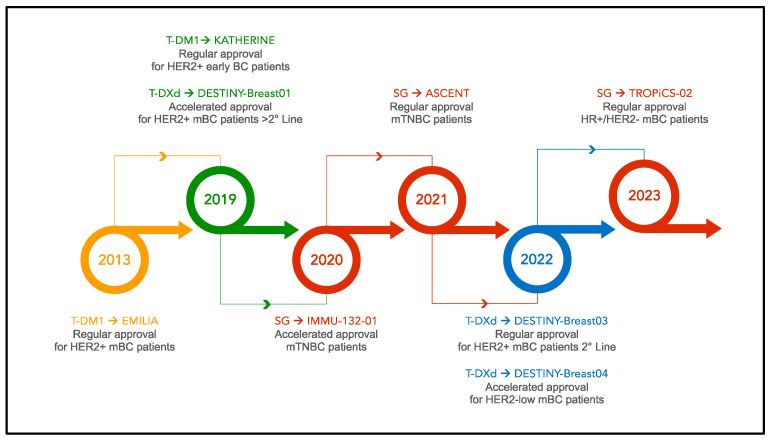
Timeline of ADC approval in breast cancer. Legend: mBC: metastatic breast cancer; mTNBC metastatic triple-negative breast cancer; SG: sacituzumab-goviteca; T-DM1: trastuzumab-emtansine; T-DXd: trastuzumab-deruxtecan.

There are currently three ADCs approved for use in BC, with several additional promising agents in development (Figure 2). The first ADC to gain Food and Drug Administration (FDA) approval in 2013 was trastuzumab-emtansine (T-DM1) for patients with metastatic HER2-positive BC based on the results of the EMILIA trial [4]. In this phase III study, HER2-positive, pretreated BC patients were randomized to receive T-DM1 or lapatinib plus capecitabine. T-DM1 consists of an anti-HER2 antibody- and a microtubule inhibitor payload connected through a non-cleavable linker. Following initial approval in the metastatic setting, subsequent approval was granted in 2019 in the adjuvant setting for patients with residual disease after neoadjuvant chemotherapy and trastuzumab, based on the KATHERINE trial [5]. A second HER2-targeting ADC, trastuzumab-deruxtecan (T-DXd), gained accelerated FDA approval in 2019 for the treatment of BC, based on data from the DESTINY Breast-01 study [6]. T-DXd has a cleavable linker and a topoisomerase I payload. Confirmatory trials of T-DXd have demonstrated remarkable improvements in patients’ outcomes in both HER2-positive and HER2-low disease [7,8]. In 2020, the FDA granted accelerated approval to sacituzumab-govitecan (SG) for patients with triple-negative breast cancer (TNBC), followed by regular approval in 2021 based on the ASCENT trial results, showing improvement in progression-free (PFS) and overall survival (OS) [9]. Sacituzumab-govitecan encompasses a TROP-2-directed antibody, a cleavable linker, and a topoisomerase-1 inhibitory payload [10]. In 2023, SG was also granted approval for hormone receptor (HR) positive metastatic BC based on results from the TROPiCS-02 trial [11].

In contrast to the results achieved by ADCs in BC, immunotherapy studies have been disappointing compared to the success seen in other tumor types. Studies investigating the addition of atezolizumab to standard chemotherapy for TNBC failed to consistently demonstrate improvements in OS, leading to the eventual withdrawal of FDA approval [12,13]. However, pembrolizumab in combination with chemotherapy is now standard of care for metastatic TNBC based on the results from the Keynote 355 trial, showing improved median OS [14]. In the neoadjuvant setting, several studies have demonstrated a higher rate of pathologic complete response (pCR) when immunotherapy is combined with standard chemotherapy for patients with TNBC (64.8% vs. 51.2% in Keynote-522; 58% vs. 41% in IMPASSION031; 43.5% vs. 40.8% in NeoTRIP), and some studies have also demonstrated prolonged event-free survival (36-month EFS 84.5% vs. 76.8% in Keynote-522) [15,16,17]. Emerging data in the neoadjuvant setting for patients with HR-positive BC are also showing promise with improved pCR rates [18,19]. 

Given the mixed success of immunotherapy for BC compared to the remarkable success of ADCs, there is significant interest in combining treatments to improve outcomes for patients. Although largely thought of as a sophisticated mechanism for drug delivery, ADCs have under-recognized immune activity, providing a potential rationale for combining ADCs with immunotherapy for patients with BC.

## 3. Preclinical Evidence: Immunogenicity of ADCs

**Figure 3:**
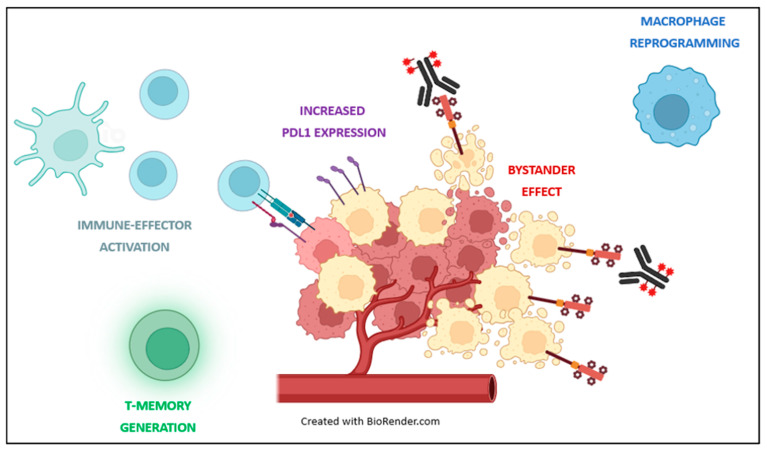
Summary of mechanism contributing to ADCs immunogenicity. Bystander effect: antitumor activity against cancer cells located near targeted antigen-positive cells, regardless of target antigen expression; immune activation: stimulation and recruitment of effective immune cells; T-memory generation: formation of memory T cells; macrophage reprogramming: alteration of macrophage cells to exhibit an anti-tumor effect; increased PD-L1: elevated expression of PD-L1, potentially leading to ICIs sensitivity.

Thanks to their structure, ADCs can have an impact not only on cancer cells but also on other elements of the TME, in particular immune cells [3] (Figure 3). In vivo studies indicate that T-cell depletion can significantly impair the anticancer efficacy of ADCs with cytotoxic payloads [4,20]. This highlights how the immunomodulatory effect of ADCs might be one of their key mechanisms of action. Designed to deliver cytotoxic drugs in the presence of a specific antigen, ADCs can result in immunogenic cell death (ICD) of targeted and surrounding cells [21]. Compared to classical apoptosis, ICD is characterized by the release of tumor-associated antigens (TAAs) and damage-associated molecular patterns (DAMPs), promoting antigen presentation by dendritic cells to T cells via the high-mobility group B1 (HMGB1)-Toll-like receptor 4 (TLR4) interaction. These molecules are recognized by dendritic cells, which uptake and process the TAAs, subsequently presenting them on major histocompatibility complex (MCH) molecules, allowing the activation of CD8+ cytotoxic T cells and CD4+ helper T cells [22,23]. The ability to induce ICD has been observed with different cytotoxic payloads, including doxorubicin, auristatin, calicheamicin, and cyclopropylpyrroloindolone, [24,25,26] supporting the immunostimulant potential of these compounds. Interestingly, microtubule depolymerizing payloads, such as vinca alkaloids, have been demonstrated to induce dendritic cell maturation, while the same property has not been observed in tubulin-stabilizing agents, such as taxanes [3,27]. Preclinical data suggest that ADCs can support long-term anti-tumor immunity by promoting the generation of T memory cells. This phenomenon is supported by several in vivo observations. D’Amico et al. used a HER2-targeting ADC with an anthracycline-based payload to treat humanized HER2-expressing (HER2+) syngeneic mice [28]. The animals who achieved a complete tumor regression were subsequently re-injected with the original HER2+ cancer cells or with a different line of tumor cells. While the mice treated with the parental HER2+ cells remained in complete remission, those who received a different line of tumor cells rapidly developed tumors. Similarly, Iwata et al. observed that syngeneic mouse models in complete response to T-DXd were able to fully reject re-inoculated cancer cells [29]. In contrast, these same tumor cells led to cancer development in unpretreated mice used as controls. Intriguingly, growing evidence indicates that ADCs might also exert their antitumor effect via interaction with innate immunity. As an example, Li et al. demonstrated that tumor-associated macrophages (TAMs) can internalize and then release membrane-permeable payloads such as auristatin or pyrrolobenzodiazepines in the TME, contributing to bystander killing of surrounding cancer cells, regardless of their target expression [30]. 

Moreover, the release of cytokines and chemokines from dying tumor cells following ADCs treatment can recruit and activate various immune cells, including macrophages and natural killer (NK) cells, which further contribute to the anti-tumor response [24]. Finally, ADCs can induce changes in cancer cells with a significant impact on their ability to escape immune surveillance. As an example, in an in vivo study, treatment with T-DXd resulted in increased expression of PD-L1 and MHC class I by cancer cells [29].

## 4. Translational Evidence: Biomarkers of Response to ADCs Alone or in Combination with Immunotherapeutic Agents

The mechanisms of action of ADCs are complex, resulting from several biological processes including—but not limited to—cellular uptake, endocytosis regulation, intracellular trafficking, activation of cellular immune defense, and sensitivity to cytotoxic agents [31,32]. Deciphering these mechanisms may disclose novel predictive biomarkers of response and resistance to ADCs that may be different from the mere expression of the target antigen. Combining ADCs with immunotherapy brings biological understanding to a further level of complexity, involving the crosstalk between tumor cells, immune cells, and other elements of the TME. Table 1 summarizes available data regarding predictive immune biomarkers of ADC activity in BC, either alone or in combination with immunotherapeutic agents.

### 4.1. Target Antigen Expression and Oncogenic Pathways

Studies have demonstrated a strong correlation between the level of expression of the target antigen and the presence of genetic mutations and the response to ADCs in BC. In an exploratory analysis of the EMILIA trial, the efficacy of T-DM1 was correlated to the expression of multiple genes (HER1, HER2, and HER3) at the mRNA level, the expression of PTEN at the protein level, and to the presence of PIK3CA point mutations [4]. PFS, but not OS benefit, was observed in patients with reduced or lost PTEN expression, treated with T-DM1. Additionally, PIK3CA mutations were associated with shorter survival in patients receiving capecitabine and lapatinib in the control arm, but not in those treated with T-DM1 in the experimental arm [33]. These results are consistent with those from the TH3RESA trial, where PFS was similar in patients receiving T-DM1 regardless of PIK3CA mutational status or PTEN expression [34]. In both trials, HER2 mRNA levels were directly correlated to treatment efficacy, both in the control and experimental arm [33,34]. More recently, evidence has been generated about biomarkers of response to third-generation ADCs, T-DXd and SG. In the phase II DAISY trial, T-DXd was tested in three cohorts of patients with advanced BC, based on HER2 expression level. Even though the magnitude of treatment benefit was directly correlated to HER2 expression, antitumor activity was observed even in HER2 0+ patients, suggesting a potential HER2-independent mechanism of action for T-DXd [35]. The phase II DESTINY-PanTumor02 trial assessed the efficacy of T-DXd across seven cohorts of patients with previously treated HER2-expressing tumors. The greatest benefit was observed in the subgroup with the highest level of HER2 expression as measured by immunohistochemistry [36]. Another biomarker analysis from the DESTINY-CRC01, including patients with advanced colorectal cancer, showed that T-DXd efficacy is correlated to higher baseline HER2 levels in both tissue (IHC/ISH) and peripheral blood (copy number of ERBB2 in plasma and circulating HER2-extra cellular domain). Furthermore, responses were observed regardless of RAS or PIK3CA mutational status and tumor mutational burden levels [37]. 

In contrast to the clear correlation between the levels of HER2 antigen expression and responses to T-DM1 and T-DxD, correlation between Trop-2 antigen expression and response to SG has not been found. The final analysis of the ASCENT trial, assessing SG in patients with metastatic TNBC (mTNBC), demonstrated improved clinical outcomes across all levels of Trop-2 expression [38]. Similarly, in the TROPICS02 trial, evaluating SG in HR+/HER2- metastatic BC patients, responses to the ADC were irrespective of Trop-2 expression levels by immunohistochemistry [11,39]. Furthermore, a mRNA-based biomarker analysis indicated that SG improved PFS and OS outcomes in patients regardless of TROP-2 mRNA levels, highlighting the need for ongoing research to identify biomarkers of response [40]. 

### 4.2. Immune Biomarkers in ADCs: Tumor-Infiltrating Lymphocytes and Neutrophil-to-Lymphocyte Ratio

Tumor-infiltrating lymphocytes (TILs) are known to influence responses to anticancer treatments. While the connection between TILs and ICIs’ success is intuitive (T cells are the main target for anti-PD-1/PD-L1 antibodies), their role in responsiveness to cytotoxic agents and ADCs is less clear [5]. TILs have been explored as potential biomarker in BC patients receiving HER2 and TROP-2-directed ADCs (Table 1). In an exploratory analysis of the EMILIA trial, TILs presence in biopsies from metastatic sites was correlated to T-DM1 treatment efficacy in 95 eligible patients. Numerically better outcomes were observed in case of TILs absence (score = 0) among patients in the control arm, while no correlation between TILs and T-DM1 activity emerged [4]. Preliminary results from the NeoSTAR trial, which evaluated neoadjuvant SG in early TNBC, showed that elevated levels of Ki-67 and TILs predicted pCR, while TROP-2 expression was not associated with tumor remission [20]. 

Although tissue-based biomarkers are still broadly used in oncology, efforts are being made to identify non-invasive biomarkers to predict prognosis and inform treatment decisions with reduced risks for patients. The neutrophil-to-lymphocyte ratio (NLR) is an example of blood-based biomarker extensively explored in patients receiving different types of anticancer treatments, in particular immunotherapy. Imamura et al. evaluated the role of NLR measured at baseline and after one cycle of treatment in 53 patients with advanced/metastatic BC, treated with T-DM1. They observed a significant correlation between low NLR at baseline and better PFS (HR 0.23, *p* = 0.0001) and OS (HR 0.38, *p* = 0.0296) in this population. In their analysis, the effect of NLR at baseline on patients’ outcomes was independent from the number of prior treatment lines, presence of visceral metastases, HER2 expression, and hormone receptor status [21]. Similarly, Li et al. used pre-treatment NLR and LDH values to generate a score to predict benefit from T-DM1 in 51 patients with HER2 positive advanced BC [22]. They reported an association between elevated NLR and high LDH levels and poor prognosis. The median PFS of patients with baseline NLR low was 7.1 months versus 4.6 months in patients with baseline NLR high (*p* = 0.003). Despite hypothesis-generating, these studies present several limitations, including the low number of patients included and the retrospective nature of the analysis. Large prospective studies are needed to confirm the role of NLR in this setting.

### 4.3. Predictive Biomarkers for ADC-Immunotherapy Combinations

Data on predictive biomarkers of response to combinations of ADCs and immunotherapeutic agents are currently limited. Research efforts have been mainly focused on immune biomarkers, given the potential impact of these combinations on immune system modulation [20].

In a phase I study evaluating the combination of T-DM1 and pembrolizumab in 20 patients with HER2-positive metastatic BC, several immune biomarkers were tested by immunohistochemistry, whole-exome sequencing (WES), and bulk RNA sequencing (RNA-seq). Some of these markers, including TILs, CD8, PD-L1, and HLA-ABC expression, showed dynamic changes over time, with an early and sustained increase over the course of therapy. Similarly, expression of genes associated with antigen presentation increased during treatment. These findings support a potential immune-activating effect of the combination regimen. Surprisingly, a lower overall response rate (ORR) was reported in PD-L1-positive patients with a combined positive score >1 or ≥10 and in subjects with high TILs (≥10%). However, these results may be due to the small sample size [23]. 

Another phase I trial tested the combination of T-DM1 with atezolizumab in patients with HER2-positive BC [24]. Correlative analyses evaluated a broad range of biomarkers, including PD-L1, TILs, gene expression signatures, and RNA-based T-cell receptor (TCR) sequencing. In patients with early or advanced BC, PD-L1 expression in immune cells increased during treatment with T-DM1 plus atezolizumab, but this finding was not correlated with tumor response. Similarly, increases in CD8+ T cells and changes in TCR clonality were observed in patients treated with the combination in the neoadjuvant setting but were not associated with pathological complete response. In the same setting, various RNA-based immune signatures increased over the course of treatment. However, these changes were not observed among patients with advanced BC [24]. 

The combination of T-DM1 and atezolizumab was further tested in the phase II KATE 2 trial, where patients with metastatic HER2+ BC were randomized (2:1) to receive either T-DM1 plus atezolizumab or T-DM1 plus placebo. A prespecified exploratory analysis of this study examined the role of different biomarkers at baseline, including TILs, PD-L1, CD8, HER2 expression and amplification, tumor mutational burden (TMB), effector T-cell gene signature, and PIK3CA mutational status. In the subgroup defined as positive for PD-L1, treatment with atezolizumab plus T-DM1 showed a trend towards improved PFS compared to T-DM1 plus placebo. In contrast, in the PD-L1 negative subgroup, no significant difference in median PFS was observed between the two arms. In the PD-L1 positive subgroup, a higher percentage of patients treated with atezolizumab achieved an objective response compared to those treated with placebo (54% versus 33%, respectively). However, in the PD-L1 negative subgroup, the ORR was lower in the atezolizumab group. This comprehensive analysis demonstrated a potential correlation between elevated immune markers and prolonged PFS, suggesting that the efficacy of T-DM1 plus atezolizumab may depend on pre-existing immunity. However, the actual extent of this benefit remains uncertain due to limited sample size and the exploratory nature of the analysis [24]. Among patients in the BEGONIA trial treated with Durvalumab plus datopotamab-deruxtecan (Dato-DXd) for first line mTNBC, patients were enrolled regardless of PD-L1/TROP-2 expression, and durable treatment responses were seen irrespective of PD-L1 expression [26]

## 5. Clinical Evidence: Trials of ADCs and Immunotherapy Combinations

### 5.1. Evidence from Clinical Trials

Based on the available preclinical and translational evidence, combining ADCs with immunotherapeutic agents represents an appealing strategy to treat BC, and several trials have been conducted or are currently underway to test whether this approach is effective and feasible in practice [27] (Table 2 and Table 3).

T-DM1 has been tested in combination with ICIs in phase I/II trials, with currently ongoing phase III studies.

A small phase Ib trial assessed the safety and efficacy of T-DM1 and pembrolizumab in 20 patients with metastatic HER2+ BC. Overall response rate was 20%, with four partial responses, while 30% of patients had stable disease (SD) for >24 weeks. Median PFS was 9.6 months (95% CI 2.8–16.0 months), with a median duration of response of 10.1 months (95% CI 3.1–not reached) [23]. Another phase 1b trial tested TDM-1 in combination with atezolizumab in patients with HER2+ BC both in the advanced and early (neoadjuvant) setting. In this study, ORR in patients with advanced BC was 35%, while 70% of patients with early-stage BC achieved a pathological complete response [24].

The same combination of T-DM1 and atezolizumab was explored in a randomized, placebo-controlled phase II trial (KATE 2), which recruited 202 patients with advanced HER2 positive BC, previously treated with trastuzumab and a taxane. The addition of atezolizumab to T-DM1 did not improve PFS in the overall population. However, a numerical trend in favor of the combination was observed in the PD-L1 positive subgroup (HR for PFS 0.60; 95% CI 0.32–1.11; *p* = 0.099). Overall survival is still immature and deserves longer follow-up [25].

The results observed in the PD-L1 positive population of the KATE 2 trial fostered the design of 2 ongoing randomized phase III studies in the advanced and early setting, respectively. The KATE 3 trial is randomizing patients with advanced HER2+/PD-L1 positive BC, previously treated with trastuzumab and a taxane, to T-DM1 and atezolizumab or T-DM1 and placebo [41]. The ASTEFANIA trial is testing the same combination in the adjuvant setting in HER2+ patients with invasive residual disease after neoadjuvant therapy [42].

T-DxD is also being tested in combination with ICIs. In the phase Ib DS8201-A-U105 trial, patients with HER2-expressing (i.e., HER2+ or HER2low) metastatic BC are treated with T-DXd plus nivolumab after standard of care failure. Preliminary data demonstrated an ORR of 66% and 50% in the HER2+ and HER2low population, respectively, while median PFS was 11.6 months (95% CI 6.9–not reached) and 7.0 months (95% CI 2.3–10.8) for HER2+ and HER2-low BC patients, respectively. These results suggest scarce, if any, benefit from the addition of nivolumab to T-DXd in this pre-treated population of HER2-expressing BC patients [43]. Another phase Ib study is ongoing, exploring the combination of T-DXd and pembrolizumab in patients with advanced HER2-expressing metastatic BC (NCT04042701).

The multi-arm BEGONIA trial includes patients with advanced HER2-low TNBC who are treated with T-DXd plus durvalumab in the first-line setting. Among the first 56 patients, ORR was 57% and median PFS was 12.6 months (95% CI 8.3–not reached) [44]. 

Datopotomab-deruxtecan (dato-DXd), an anti TROP-2 ADC linked to a topoisomerase I inhibitor payload, is also being tested in combination with immunotherapy. In a separate arm of the BEGONIA trial, patients with mTNBC patients regardless of HER2 status are being treated with dato-DXd and durvalumab. Data from the first 47 patients enrolled showed an impressive 79% ORR, including 2 complete and 32 partial responses, with 96% of patients maintaining the response at the time of data cut-off [45].

Sacituzumab-govitecan is also being tested in combination with immunotherapy. The randomized phase II trial SACI-IO trial will determine whether, in the first-line setting, adding pembrolizumab to SG could prolong PFS of PD-L1 negative TNBC patients [46]. In the non-metastatic setting, the phase III ASCENT-05/OptimICE-RD trial aims to enroll more than 1500 TNBC patients with invasive residual disease after neoadjuvant chemo-immunotherapy and randomize them to pembrolizumab plus SG or treatment of physician’s choice (pembrolizumab +/− capecitabine). Results from both of these trials are awaited [26].

Lastly, the combination of ladiratuzumab vedotin, an anti-LIV-1 antibody conjugated with a monomethyl auristatin E payload, and pembrolizumab is currently under evaluation in a phase 1b/2 trial enrolling patients with advanced TNBC in the first-line setting. Preliminary results from 26 patients demonstrated encouraging signs of activity for this regimen, with an ORR of 54% [47].

**Table 2:** Results from clinical trials exploring ADC-ICI combinations.

ADC	ICI	Trial Name	Phase	Study Design	Study Population (n° Enrolled)	Primary End Point	Efficacy Outcomes	Toxicity Outcomes	Ref.
T-DM1	Atezolizumab	Hamilton et al. (Cohort 1B)	Ib	T-DM1 + Atezolizumab	mBC, HER2+ (6)	Safety and tolerability	ORR 35%	Any grade 100% G3–G4 50% sAE 33%	[24]
		Hamilton et al. (Cohort 2C)	Ib	T-DM1 + Atezolizumab	mBC, HER2+ (14)	Safety and tolerability	ORR 35%	Any grade 100% G3–G4 71% sAE 57%	[24]
		Hamilton et al. (Cohort 2B)	Ib	T-DM1 + Atezolizumab (Neodjuvant)	eBC, HER2+ (20)	Safety and tolerability	pCR 70%	Any grade 100% G3–G4 80% sAE 20%	[24]
		KATE 2	II	T-DM1 + Atezolizumab vs. T-DM1 + placebo	mBC, HER2+ (202)	PFS	PFS 8.2 vs. 6.8 months	sAE 19% G5 1	[25]
		IMpassion050	III	T-DM1 + Atezolizumab vs. T-DM1 + placebo (post-neodjuvant phase)	eBC, HER2+ (454)	pCR (neoadjuvant phase) in ITT and PD-L1+	NA	Any grade 96.6% G3–G4 25.9% sAE 8.6%	[48]
	Pembrolizumab	Waks et al.	Ib	T-DM1 + Pembrolizumab	mBC, HER2+ (20)	Safety and tolerability	ORR 20% CBR 50% PFS 9.6 months DOR 10.1 months	Any grade 85% G3 20% sAE 10%	[23]
T-DXd	Durvalumab	BEGONIA (Arm 6)	Ib/II	T-DXd + Durvalumab	mTNBC (56)	Safety and tolerability	ORR 57% PFS 12.6 months	Any grade 36% sAE 9%	[44]
	Nivolumab	DS8201-A-U105	Ib	T-DXd + Nivolumab	mBC, HER2+ (48)	ORR	ORR 65.6% ORR (HER2-low) 50% PFS 11.6 months PFS (HER2-low) 7 months	G3–G4 50%	[43]
Dato-DXd	Durvalumab	BEGONIA (arm 7)	Ib/II	Dato-DXd + Durvalumab	mTNBC (47)	Safety and tolerability	ORR 79%	Any grade 36% sAE 15%	[44]
Ladiratuzumab vedotin	Pembrolizumab	Han et al.	Ib/II	Ladiratuzumab-vedotin + Pembrolizumab	a/mBC, HR−/HER2− (51)	Safety, tolerability, and activity	ORR 54%	Any grade 86%	[47]

Table legend: ADC: antibody drugs conjugate; a/mBC: advanced or metastatic breast cancer; CBR: clinical benefit rate; dato-DXd: datopotamab-deruxtecan; DOR: duration of response; eBC: early-stage breast cancer; HR: hormone receptor; ICI: immune-checkpoint inhibitor; ITT: intent-to-treat; mBC: metastatic breast cancer; mTNBC: metastatic triple-negative breast cancer; NA: not available; ORR: objective response rate; pCR: pathologic complete response; PFS: progression-free survival; sAEs: serious adverse events; T-DM1: trastuzumab-emtansine; T-DXd: trastuzumab-deruxtecan.

**Table 3:** Ongoing clinical trials exploring ADC-ICI combinations.

ADC	ICI	Trial Name	NCT	Phase	Study Design	Study Population (Expected Enrollment)	Primary End Point
T-DM1	Atezolizumab	KATE 3	NCT04740918	III	T-DM1 + Atezolizumab vs. T-DM1 + placebo	mBC, HER2+, PD-L1+ (96)	PFS, OS
		ASTEFANIA	NCT04873362	III	T-DM1 + Atezolizumab vs. T-DM1 + placebo	eBC, HER2+ without pCR after NAT (1700)	IDFS
T-DXd	Pembrolizumab	NA	NCT04042701	Ib	T-DXd + Pembrolizumab	mBC, HER2+ (115 *)	DLT/MTD; ORR
	Durvalumab	DESTINY-Breast07	NCT04538742	Ib/II	T-DXd + Durvalumab (Module 1)	mBC, HER2+ (244 in total)	Safety
		DESTINY-Breast08	NCT04556773	Ib	T-DXd + Durvalumab + Paclitaxel (Module 2)	HER2-low mBC (139 in total)	Safety
		TRUDI	NCT05795101	II	T-DXd + Durvalumab	eBC HER2-expressing, neoadjuvant (63)	pCR
SG	Pembrolizumab	SACI-IO	NCT04468061	II	SG + Pembrolizumab vs. SG	mTNBC (110)	PFS
		ASCENT-05 OptimICE-RD	NCT05633654	III	SG + Pembrolizumab vs. Pembrolizumab ± Capecitabine	TNBC without pCR after NAT (1514)	IDFS
	Atezolizumab	ASPRIA	NCT04434040	II	SG + Atezolizumab	TNBC without pCR after NAT (40)	ctDNA clearence at 18 weeks
	Avelumab	InCITe	NCT03971409	II	SG+ Avelumab (Arm B)	mTNBC (150 in total)	ORR
Dato-DXd	Durvalumab	I-SPY 2	NCT01042379	II	Dato-DXd + Durvalumab (multiarm, adaptive trial)	eBC (5000 in total)	pCR
		TROPION-03	NCT05629585	III	Dato-DXd + Durvalumab vs. Dato-DXd vs. Pembrolizumab and/or Capecitabine	TNBC without pCR after NAT (1075)	IDFS
		TROPION-04	NCT06112379	III	Dato-DXd + Durvalumab vs. Pembrolizumab + CHT	TNBC; HR-low/HER2−, neoadjuvant (1728)	pCR; EFS
		NA	NCT06103864	III	Dato-DXd ± Durvalumab vs. CHT + Pembrolizumab	mTNBC; PD-L1+ (625)	PFS

Table legend: CHT: chemotherapy; dato-DXd: datopotamab-deruxtecan; DLT: dose-limiting toxicity; eBC: early-stage breast cancer; EFS: event-free survival; HR: hormone receptor; IDFS: invasive disease-free survival; mBC: metastatic breast cancer; MTD: maximum tolerated dose; mTNBC: metastatic triple-negative breast cancer; NA: not available; NAT: neoadjuvant therapy; ORR: objective response rate; OS: overall survival; pCR: pathologic complete response; PFS: progression-free survival; SG: Sacituzumab-govitecan; T-DM1: trastuzumab-emtansine; T-DXd: trastuzumab-deruxtecan; TNBC: triple-negative breast cancer; * including non-small cell lung cancer patients.

### 5.2. Toxicity Profile of ADC and Immunotherapy Combination

Combining anticancer agents offers the opportunity to enhance their efficacy, but it also increases the risk of developing toxicities [2]. Adverse events related to ADCs vary according to the specific agents and may include hematological toxicity, hepatic toxicity, interstitial lung disease, peripheral neuropathy, and ocular disease, among others [49]. The toxicity profile of ICIs is characterized by the onset of immune-related adverse events (irAE) of variable grades due to hyperactivation of patients’ immune system. Common manifestations include thyroiditis, dermatitis, pneumonitis, and gastrointestinal disorders, but any organ can be involved [50]. 

Given the broad spectrum of adverse events reported with either ADCs or immunotherapeutic agents, toxicity represents a potential concern for the development of combination regimens. However, available data from clinical trials do not seem to suggest significant signs of overlapping side effects (Table 2).

The combination of T-DM1 and ICIs has been evaluated in several studies. In non-randomized, early-phase clinical trials testing T-DM1 plus atezolizumab or pembrolizumab, the most frequent adverse events (AE) of any grade included fatigue, diarrhea, anemia, and nausea [23,24]. Over 50% of patients treated with T-DM1 plus atezolizumab encountered at least one grade (G) ≥ 3 AE. Notably, this incidence was particularly pronounced in the early-stage setting, reaching an 80% incidence rate. T-DM1 in combination with pembrolizumab reported a lower occurrence of G ≥ 3 AEs, with an incidence rate of 30%. No G5 toxicities were registered in these studies. In the randomized KATE 2 trial, the incidence of G ≥ 3 AE was comparable between the control and experimental arm and included thrombocytopenia, increased aspartate aminotransferase, anemia, neutropenia, and increased alanine aminotransferase. However, treatment discontinuation due to adverse events was more frequent in patients receiving T-DM1 plus atezolizumab compared to T-DM1 alone, and one treatment-related death occurred in the experimental group due to hemophagocytic syndrome [25]. In the post-neoadjuvant phase of the IMPASSION030 trial, patients with invasive residual disease after pre-operative chemo-immunotherapy with trastustuzumab, pertuzumab, atezolizumab, or placebo received T-DM1 plus atezolizumab or placebo. In this population, the rate of AE was comparable between the experimental and control arms in terms of G ≥ 3 toxicities [48]. 

An interim analysis of the BEGONIA trial exploring durvalumab plus T-DXd or dato-DXd identified nausea (55%), stomatitis (51%), and diarrhea (13%) as common AEs. Adverse events G ≥ 3 were experienced by 36% of patients receiving both combinations, but no toxicity-related deaths were observed [46,51]. As for the combination of T-DXd with nivolumab, in the DS8201-A-U105 trial the most frequent toxicities were nausea (56.3%) and interstitial lung disease (14.6%) [43].

Lastly, combining ladiratuzumab with pembrolizumab determined enhanced gastrointestinal toxicity, with colitis being the most common G3 adverse event [47].

## 6. Conclusions

Preclinical and clinical evidence suggests that by harnessing targeted cytotoxicity and immune modulation, ADCs not only deliver potent payloads directly to tumor cells but also engage the host immune system. This engagement leads to the activation of immune responses against tumor antigens, orchestrating a multifaceted antitumor response that surpasses traditional chemotherapy’s scope [28,52]. This synergistic interaction between targeted cytotoxicity and immune activation holds promise for overcoming tumor heterogeneity, reducing treatment resistance, and improving patient outcomes.

In the era of precision oncology, the quest for biomarkers to guide therapeutic decisions has assumed paramount importance. Immune biomarkers may hold predictive value for ADC therapy, given the immunostimulant activity demonstrated by this class of agents [2]. While early findings are encouraging, further validation studies and prospective clinical trials are needed to elucidate the role of these biomarkers in predicting response to ADC therapy and guiding treatment decisions in BC patients. A potential pitfall in the exploration of biomarkers of response to ADC in the preclinical setting may be the choice of the proper in vivo or ex vivo model. Indeed, models that preserve TME and immune competence should be preferred in this scenario [53].

Early-phase clinical trials investigating ADC-ICI combinations have shown promising results, suggesting a potential synergistic effect between these two modalities [24]. Despite the therapeutic potential of ADC-ICI combinations and the apparently manageable toxicity profile that emerged from clinical studies thus far, overlapping toxicities remain a concern and require careful monitoring. The limited data on the safety profile and long-term outcomes of these combination regimens highlight the need for continued pharmacovigilance and real-world evidence generation to inform clinical practice and optimize patient care.

Many other questions need to be addressed, one of them is the potential effect of corticosteroids on the efficacy of ADCs. While plenty of evidence exists about the interplay between corticosteroids and ICIs [54], no data have been generated about the same topic thus far.

Ongoing and future studies will further elucidate the optimal dosing, sequencing, and patient selection criteria for ADC-ICI combinations, with the goal of improving their outcomes while preserving their quality of life.

## Figures and Tables

**Table 1:** Immune biomarkers of response and resistance to ADC alone or in combination with immune-checkpoint inhibitors.

	Biomarker	Source	Treatment	Setting	Results	Refs.
**ADC** **single agent**	TILs	Tissue	T-DM1	95 patients HER2+ mBC	- Improved PSF and OS in the absence of TILs (0) vs. presence of TILs (≥1) - No differences in PFS and OS in a higher TIL cutoff (TIL > 10) - In the T-DM1 arm, no association between TILs and PFS/OS	[4]
	TILs	Tissue	SG	50 patients eTNBC	- TILs were predictive of pCR to SG (*p* = 0.002)	[20]
	NLR	Blood	T-DM1	53 patients HER2+ mBC	- PFS benefit in NLR low group (*p* = 0.0001) - OS benefit in NLR low group (*p* = 0.0296).	[21]
	NLR	Blood	T-DM1	51 patients HER2+ mBC	- PFS benefit in NLR low group (*p* = 0.003) - OS benefit in NLR low group (*p* = <0.001)	[22]
**ADC-ICI combos**	PD-L1 and TILs	Tissue	T-DM1 + Pembrolizumab	20 patients HER2+ mBC	- ORR lower in PD-L1+ (>1 or ≥10) group and high-TILs group (≥10%) - No associations between PD-L1 status, TILs, and clinical benefits	[23]
	PD-L1, TILs and TCR-Seq	Tissue	T-DM1 + Atezolizumab	73 patients HER2+ early/mBC	- PD-L1 IC increased in patients with mBC and eBC, not associated with clinical outcomes - Increase in CD8+ T cells in patients with eBC, no association with pCR - No statistically significant changes in TCR clonotype number or diversity index	[24]
	PD-L1, TILs and CD8	Tissue	T-DM1 + Atezolizumab	330 patients HER2+ mBC	- PFS benefit in the PD-L1+ subgroup (8.8 vs. 4.1 months; HR 0.60, 95% CI 0.32–1.11) - Better PFS in high-TILs subgroup (HR for PFS: 0.62 [0.37–1.03] in patients with TILs ≥ 5% vs. 1.52 [0.76–3.04] in patients with TILs) - OS benefit in the PD-L1+ subgroup (1-year rate 94.3% vs. 87.9%, HR 0.55, 95% CI 0.22–1.38) - T-cell activation biomarkers enriched in the PD-L1+ subgroup	[25]
	PD-L1	Tissue	Dato-DXd + Durvalumab	62 patients mTNBC	- Response to treatment independent of PD-L1 expression level	[26]

Table legend: ADC: antibody-drug conjugate; Dato-DXd: datopotamab-deruxtecan; eBC: early breast cancer; eTNBC: early triple-negative breast cancer; ICI: immune-checkpoint inhibitor; mBC: metastatic breast cancer; mTNBC: metastatic triple-negative breast cancer; NLR: neutrophil-to-lymphocyte ratio; ORR: objective response rate; OS: overall survival; pCR: pathologic complete response; PFS: progression-free survival; SG: sacituzumab-govitecan; T-DM1: trastuzumab-emtansine; TCR: T-cell receptor; TILs: tumour-infiltrating lymphocytes.

## Data Availability

All data generated or analyzed during this study are included in this article. Further inquiries can be directed to the corresponding author.
